# YY1: a key regulator inhibits gastric cancer ferroptosis and mediating apatinib-resistance

**DOI:** 10.1186/s12935-024-03262-z

**Published:** 2024-02-12

**Authors:** Zi-Han Geng, Jun-Xian Du, Yue-Da Chen, Pei-Yao Fu, Ping-Hong Zhou, Wen-Zheng Qin, Yi-Hong Luo

**Affiliations:** 1 Department of General Surgery, Zhongshan Hospital, Fudan University, 200032 Shanghai, China; 2 Department of General Surgery, Zhongshan Hospital, Fudan University (Xiamen Branch), 361004 Xiamen, Fujian China; 3 Endoscopy Center and Endoscopy Research Institute, Zhongshan Hospital, Fudan University, 200032 Shanghai, China; 4 Shanghai Collaborative Innovation Center of Endoscopy, 200032 Shanghai, China

**Keywords:** Gastric cancer, YY1, Ferroptosis, Apatinib, p53

## Abstract

**Objective:**

Gastric cancer (GC) stands as a prevalent and deadly global malignancy. Despite its role as a preoperative neoadjuvant therapy, Apatinib’s effectiveness is curtailed among GC patients exhibiting elevated YY1 expression. YY1’s connection to adverse prognosis, drug resistance, and GC metastasis is established, yet the precise underlying mechanisms remain elusive. This study aims to unravel potential pathogenic pathways attributed to YY1.

**Design:**

Utilizing bioinformatics analysis, we conducted differentially expressed genes, functional annotation, and pathway enrichment analyses, and further validation through cellular and animal experiments.

**Results:**

Higher YY1 expression correlated with diminished postoperative progression-free survival (PFS) and disease-specific survival (DSS) rates in TCGA analysis, identifying YY1 as an independent DSS indicator in gastric cancer (GC) patients. Notably, YY1 exhibited significantly elevated expression in tumor tissues compared to adjacent normal tissues. Bioinformatics analysis revealed noteworthy differentially expressed genes (DEGs), transcriptional targets, factors, and co-expressed genes associated with YY1. LASSO Cox analysis unveiled Transferrin as a prospective pivotal protein regulated by YY1, with heightened expression linked to adverse DSS and PFS outcomes. YY1’s role in governing the p53 signaling pathway and ferroptosis in GC cells was further elucidated. Moreover, YY1 overexpression dampened immune cell infiltration within GC tumors. Additionally, YY1 overexpression hindered GC cell ferroptosis and mediated Apatinib resistance via the p53 pathway. Remarkably, IFN-a demonstrated efficacy in reversing Apatinib resistance and immune suppression in GC tissues.

**Conclusions:**

Our findings underscore the pivotal role of YY1 in driving GC progression and influencing prognosis, thus pinpointing it as a promising therapeutic target to enhance patient outcomes.

**Supplementary Information:**

The online version contains supplementary material available at 10.1186/s12935-024-03262-z.

## Introduction

Gastric cancer (GC) is a prevalent malignant tumor with a high incidence and poor prognosis. According to the latest epidemiological research and statistics, GC ranks sixth globally in terms of incidence and third in terms of mortality [1]. Additionally, GC exhibits an insidious onset and a high morbidity rate. Approximately half of GC patients lose the opportunity for radical surgery due to locally advanced disease or distant metastasis at the time of treatment, consequently resulting in a high mortality rate. Survival data across different regions for GC indicates that, as the tumor progresses in stages, the 5-year survival rate gradually decreases, with a more pronounced decline observed after stage IIIb [2]. Apart from enhancing early diagnosis through gastroscopy screening, the first-diagnosed advanced GC patients can only improve their prognosis by undergoing multidisciplinary comprehensive treatments [3].

In our previous clinical practice and research, we have observed significant overexpression of YY1 in GC patients who exhibited poor response to Apatinib during preoperative neoadjuvant therapy. Even in cases where Apatinib was administered alongside XELOX, around 30% of patients demonstrated resistance to the treatment (JGCA TRG 0-Ia). Apatinib, functioning as a small molecule tyrosine kinase inhibitor, effectively reduces VEGF-mediated endothelial cell migration and proliferation. It exerts its anti-tumor effects by selectively binding to and inhibiting the activation of vascular endothelial growth factor receptor 2 (VEGFR-2), consequently impeding tumor micro-angiogenesis. This mechanism not only enhances the efficacy of chemotherapeutic drugs [4], but also sensitizes immune checkpoint inhibitors to a certain extent [5–7]. This makes Apatinib the world’s first safe and effective small-molecule anti-vascular drug for advanced GC treatment. Despite these advancements, challenges related to drug resistance and acquired resistance to targeted therapies remain pertinent. Furthermore, due to the high heterogeneity among patients, responses can vary widely. For instance, the objective response rate (ORR) of Apatinib is recorded at 1.7% (compared to 0% in the placebo group), while the disease control rate stands at 31.82% (compared to 10.99% in the placebo group) [8]. Additionally, long-term treatment may lead to acquired drug resistance, significantly impacting the prognosis of GC patients in China.

Many types of cancer-related cells, including B-cell lymphoid tumors, follicular lymphomas, acute myeloid leukemia, gastric cancer, osteosarcoma, cervical cancer, brain tumors, prostate cancer, colon cancer, ovarian cancer, breast cancer, and lung cancer cells, exhibit a high expression of YY1 [9]. Furthermore, numerous studies have demonstrated a significant correlation between YY1 expression and poor prognosis, drug resistance, as well as cancer metastasis. YY1 plays a substantial role in the progression of multiple tumor types, albeit with varying mechanisms through which it promotes tumor growth. Interestingly, YY1 displays a dual function in transcriptional regulation and tumor growth, functioning both as an activator and a suppressor [10]. This duality underscores the complexity of its role. Consequently, gaining a comprehensive understanding of the mechanisms underlying YY1’s function as both a tumor promoter and suppressor is of paramount importance. Such insights hold the potential to guide the development of novel therapeutic strategies targeting YY1 for effective tumor therapy.

## Methods

### Access to TCGA datasets, data normalization, and analysis of differentially expressed genes (DEGs)

First, we retrieved the expression profiles and downloaded the v22 version ( (https://www.gencodegenes.org/human/release_22/gencode.v22.annotation.gff3.gz) file and the v33 version (http://ftp.ebi.ac.uk/pub/databases/gencode/Gencode_human/release_33/gencode.v33.annotation.gff3.gz) of the gff3 files from GENCODE http://ftp.ebi.ac.uk/pub/databases/gencode/Gencode_human) [11]. Then, we extracted the mapping information of GeneSymbol and ENSG_ID, using the map ENSG_ID to GeneSymbol function. When multiple matches were detected, the median was retrieved, and the converted expression profile was finally obtained.

We first employed normalized gene expression data, segregating patients into high and low YY1 expression groups based on the median expression level of YY1. To identify the DEGs between control and different comparison groups, differential expression analysis was conducted using the “limma” R package [12]. Specifically, for the expression profile dataset obtained, the proportion with an expression value of 0 greater than 50% of the genes was removed, and the “voom” function was used for data transformation. Next, we used the “lmFit” function for multiple linear regression, and the “eBays” function to calculate the moderated t- and F-statistics. We also used the log-odds of differential expression by empirical Bayes moderation of the standard errors towards a common value, resulting in a significant difference for each gene. Finally, we obtained the significant difference of each gene, resulting in the DEGs between high and low YY1 expression groups.

### Functional annotation and pathway enrichment analyses

The transcriptional targets and transcription factors related to YY1 were obtained from TRRUST v2 [13] (https://www.grnpedia.org/trrust/). We used GO annotations via the “org.Hs.eg.db” (version 3.1.0) R package. The latest gene annotation of KEGG pathways, used as a background, was obtained using the KEGG rest API (https://www.kegg.jp/kegg/rest/keggapi.html). Then, the “clusterProfiler” R package was used to map the genes into the background set, and the enrichment analysis was conducted to evaluate the enrichment of the gene set. The gene set number was defined between 5 and 5000, and significant differences were defined by an FDR < 0.25 and *p* < 0.05. Next, we performed gene set enrichment analysis (GSEA) using the GSEA software (version 3.0; http://software.broadinstitute.org/gsea/index.jsp). We predefined the gene ranking and used the c2.all.v7.4.symbols.gmt sub-collection to assess related pathways and molecular mechanisms. Based on a predetermined gene ranking, the gene set number was defined between 5 and 5000 and one thousand resamplings were performed. Finally, significant differences were defined by an FDR < 0.25 and *p* < 0.05.

### Evaluation of immune cell infiltration

Furthermore, we calculated the stromal for each patient in each tumor group based on the gene expression profile using the “ESTIMATE” R package [14]. The infiltration of immune cells was analyzed using ssGSEA. Further, to evaluate the correlation coefficients between immune cell infiltration and YY1 expression, we used the Tumor IMmune Estimation Resource (TIMER 2.0) [15–17].

### Construction of YY1-overexpressed and SLC7A11-knockdown cells and transfection

The YY1-overexpressed and SLC7A11-knockdown human HGC-27 GC and mice MFC GC cells were purchased from Merdobio Co. Ltd. and the Cell Bank of the Institute of Biochemistry and Cell Biology, respectively. Cells were cultured in FBS (10%, Biotime)-supplemented DMEM medium (Biotime) [18]. Lentivirus packaging was performed by Merdobio Co. Ltd. (Shanghai) and cells were transfected with Lipofectamine (Life) as previously described. Briefly, target cells were infected with filtered lentivirus plus 6 µg/mL polybrene to generate stable cell lines. Then, MFC and HGC-27 cells were infected with viruses, and puromycin was added for selection.

### Cell proliferation, viability, and invasion assays

First, the CCK-8 solution (20 µL, Dojindo) was added to each well of cells (1000 cells/100 µL medium) at the indicated time points to evaluate cell growth and viability. Then, 10^6^ GC cells were added into the upper chamber membrane pre-coated with Matrix Gel, and 10% FBS-containing DMEM was added into the lower chamber, as previously described [19]. After 48 h of incubation, the invaded cells were fixed, stained, and counted to evaluate the invasion capacity.

### Measurement of iron concentration and oxidative stress

The concentrations of total iron and intro cellular Fe^2+^ and GSH were detected using an iron assay kit from Merdo Bio Inc. (Shanghai, China) and Abcam. To detect changes in the oxidative stress of cells, the malondialdehyde concentration was determined using commercial kits (Nanjing Jiancheng).

### Western blot and qRT-PCR

After extraction using Trizol and quality control, RNA was reversely transcribed to cDNA using the PrimeScript™ RT reagent Kit (Takara). Then, the qRT-PCR was conducted using the SYBR® Premix Ex Taq™ II (Takara) to quantify the expression of the target gene. The protein level changes were determined by Western blot, as previously described [20].

### Luciferase reporter assay

Luciferase reporter assay Transferrin 3′UTR was cloned into the pGL3 plasmid (Promega WI, US) as previously described [21]. Cells with lentivirus-YY1 and vector were cotransfected with pGL3-YY1 or pRL-TK vector using Lipofectamine 3000 (Life Technologies, MD). Twenty-four hours after transfection, the luciferase activity was measured and normalized to the vector according to the protocol as previously described [21].

### Animal assays

Male nude mice (6 weeks) were obtained from the SLAC ANIMAL (Shanghai, China) and housed in specific-pathogen-free (SPF) conditions [22]. During experiments, food and water were freely accessed by the animals. All procedures, operations, and protocols were carefully reviewed by The Research Ethics Committee of Zhongshan Hospital and approved. For the murine xenograft model, 3 × 10^6^ lentivirus-infected cells were subcutaneously injected into mice. After 5 weeks of injection, the mice were killed and tumor tissues were collected and resected for further analysis. For tumor therapy, mIFN-α formulation (1 × 10^4^ IU, Miltenyi Biotec; #130-093-130) or anti-PD-1 antibody (10 mg/kg, Bio X Cell, BE0061, RRID: AB_1125541) were intraperitoneally injected every day. The isotype control, mAb (BioXCell, BE0089), was used as a negative control. After 6 weeks of tumor inoculation, the tumor tissues were collected for further histological analysis [23, 24].

### Immunohistochemistry (IHC) detection

The IHC was performed as previously described [25]. The antibodies used for IHC were anti-CD27 (Abcam Cat# ab214043, 1:100), anti-CD8 (Abcam Cat# ab109228, 1:100), anti-CD19 (Abcam Cat# ab245235, 1:100), anti-PD-L1 (Abcam Cat# ab213480, 1:100), and anti-YY1 (Abcam Cat# ab109228, 1:250). The scores of the IHC results were blindly accessed by two pathologists [26]. Briefly, we defined the negative staining as score 0; weak staining (ex. light yellow) as score 1; moderate staining (ex. yellow-brown) as score 2; and strong staining (ex. brown) as score 3. The percentage of positively stained cells was defined as the proportion between 0 and 100%.

### Statistical analysis

All data were processed using SPSS 21.0 (SPSS Inc.). The cut-off value for high or low gene expression was determined by the median value of relative gene expression. Spearman correlation analyses were carried out to evaluate the correlations between gene expression and immune infiltration. The Fisher’s exact test or Chi-square (χ2) tests were employed for the analysis of categorical variables related to the patients’ basic characteristics. For continuous variables such as mRNA expression, relative luciferase signal, IHC score, and tumor volume, we implemented t-tests to analyze differences between two groups with normally distributed data. In the case of comparisons involving more than two independent groups, ANOVA was employed. The Kaplan-Meier curves were used to analyze survival outcomes, and the survival from indicated groups was compared using the log-rank test, and multivariate analysis based on the Cox proportional hazards method. Results were visualized using the “ggplot2” (3.3.3) R package. All statistical analyses were 2-sided and a *p* < 0.05 was considered statistically significant [21].

## Results

### The bioinformatics analysis of TCGA STAD dataset indicated that a high expression of YY1 is related to poor GC prognosis

First, we analyzed the expression of YY1in GC tumors and adjacent normal tissues and divided all enrolled patients into two groups based on their YY1 expression. The basic characteristics of patients are summarized in Table 1.

**Table 1:** Basic characteristics of patients

Characteristic	Low YY1(*n* = 187)	High YY1(*n* = 188)	*p*
Age, median (IQR)	66 (58, 73)	68 (59, 73)	0.522
Gender, n (%)			0.718
Female	69 (18.4%)	65 (17.3%)	
Male	118 (31.5%)	123 (32.8%)	
T stage, n (%)			0.066
T1	12 (3.3%)	7 (1.9%)	
T2	46 (12.5%)	34 (9.3%)	
T3	86 (23.4%)	82 (22.3%)	
T4	40 (10.9%)	60 (16.3%)	
N stage, n (%)			0.159
N0	64 (17.9%)	47 (13.2%)	
N1	48 (13.4%)	49 (13.7%)	
N2	34 (9.5%)	41 (11.5%)	
N3	31 (8.7%)	43 (12%)	
M stage, n (%)			0.398
M0	168 (47.3%)	162 (45.6%)	
M1	10 (2.8%)	15 (4.2%)	
Pathologic stage, n (%)			0.189
Stage I	30 (8.5%)	23 (6.5%)	
Stage II	59 (16.8%)	52 (14.8%)	
Stage III	69 (19.6%)	81 (23%)	
Stage IV	14 (4%)	24 (6.8%)	
Histological type, n (%)			0.387
Diffuse Type	36 (9.6%)	27 (7.2%)	
Mucinous Type	12 (3.2%)	7 (1.9%)	
Not Otherwise Specified	102 (27.3%)	105 (28.1%)	
Papillary Type	1 (0.3%)	4 (1.1%)	
Signet Ring Type	5 (1.3%)	6 (1.6%)	
Tubular Type	31 (8.3%)	38 (10.2%)	
Histologic grade, n (%)			0.897
G1	6 (1.6%)	4 (1.1%)	
G2	69 (18.9%)	68 (18.6%)	
G3	109 (29.8%)	110 (30.1%)	
Anatomic neoplasm subdivision, n (%)			0.543
Antrum/Distal	70 (19.4%)	68 (18.8%)	
Cardia/Proximal	28 (7.8%)	20 (5.5%)	
Fundus/Body	58 (16.1%)	72 (19.9%)	
Gastroesophageal Junction	22 (6.1%)	19 (5.3%)	

**Fig. 1:**
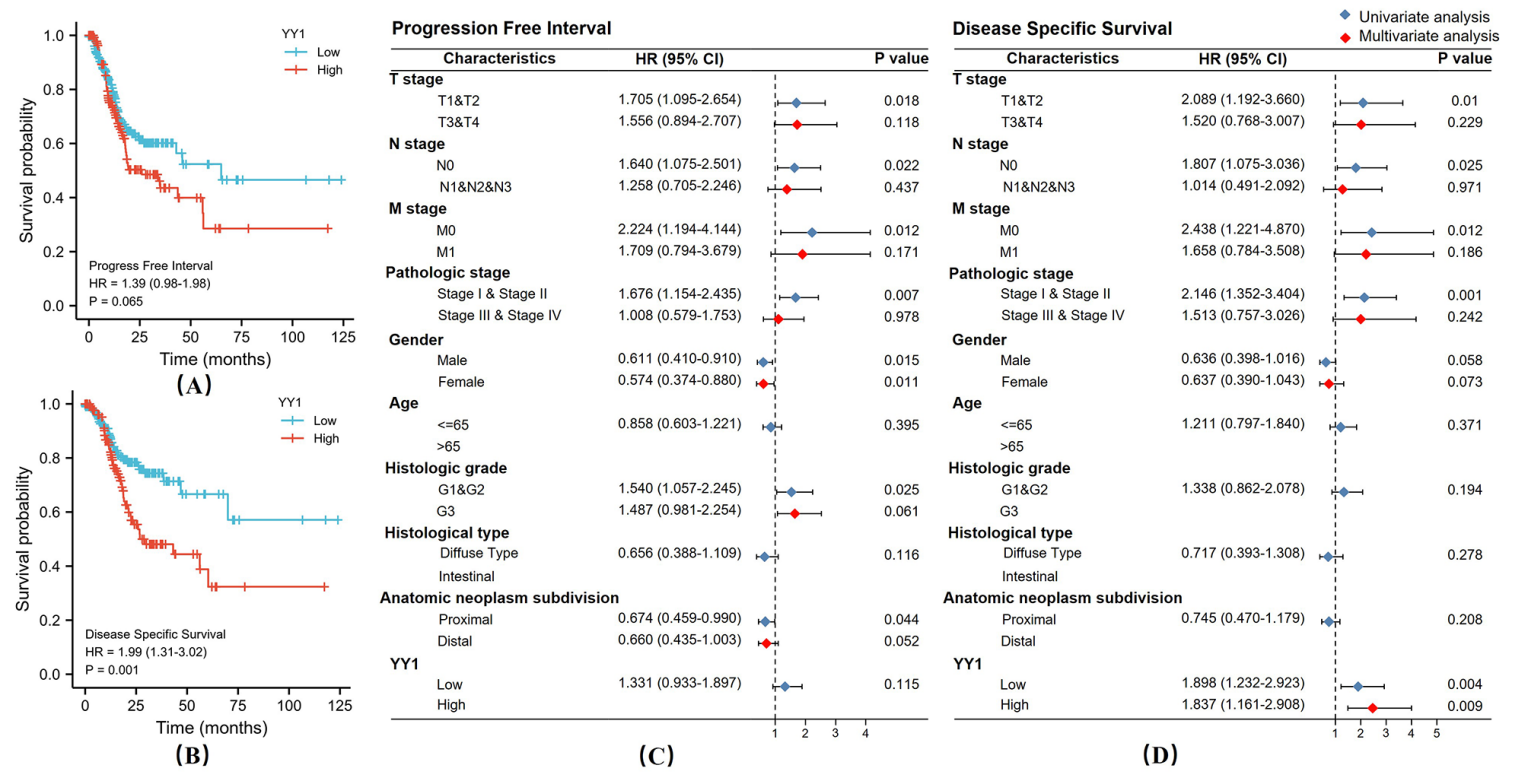
High expression of YY1 related to poor GC prognosis. **(A and B) **TCGA data showed that patients with high YY1 levels have significantly lower postoperative PFS [HR = 1.39 (0.98 − 1.98), * *p* = 0.065] and DSS [HR = 1.99 (1.31 − 3.02), **p < 0.01) rates. **(C and D)** Multivariate analysis showed YY1 expression status and correlation with DSS [HR = 1.84 (1.16, 2.91), **p < 0.01]

**Fig. 2:**
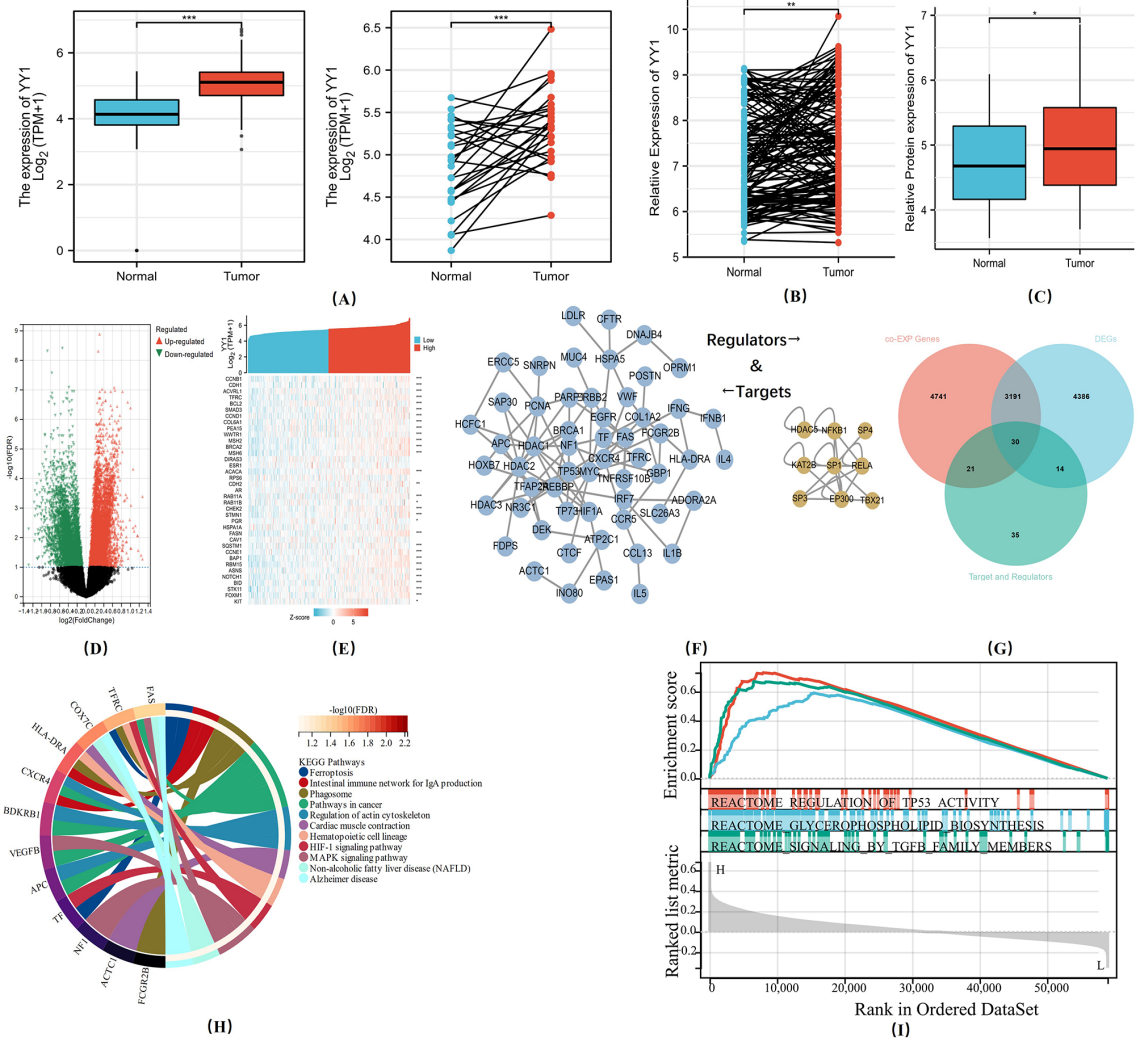
DEGs between patients with high and low YY1 expression. **(A) **YY1 expression in tumor and adjacent gastric tissues (***p < 0.001, Paired and Non-paired t test). **(B)** qRT-PCR analysis of YY1 mRNA expression and Western blot of YY1 expression **(C)** 150-paired gastric cancer and adjacent normal gastric tissues identified YY1 mRNA (**p < 0.01, Paired t test) and 50-paired tissue protein expression(*p < 0.001, Non-paired t test) showed YY1 is significantly elevated in gastric tumor tissue. **(D) **A total of 4386 significant differentially expressed genes (DEGs) were detected between GC patients with high and low YY1 expressions. **(E) **A total of 4741 YY1 co-expressed genes were downloaded from the cbioportal. **(F)** A total of 105 transcriptional targets and 11 transcription factors related to YY1 were predicted by the TRRUST v2 database. **(G)** 30 genes were identified on the Venn diagram of the intersection between DEGs, co-expressed genes, regulators, and targets of YY1. **(H)** KEGG enrichment analysis on the 30 hub genes. **(I)** GSEA combining all DEGs, co-expressed genes, regulators, and targets of YY1. TP53_ACTIVITY_THROUGH_PHOSPHORYLATION, REACTOME_SIGNALING_BY_TGFB_FAMILY_MEMBERS, and REACTOME_GLYCEROPHOSPHOLIPID_BIOSYNTHESIS were the significantly enriched pathways regulated by YY1

Furthermore, TCGA data showed that patients with higher levels of YY1 presented significant lower postoperative PFS [HR = 1.39 (0.98 − 1.98), *p* = 0.065] and DSS [HR = 1.99 (1.31 − 3.02), ** *p* < 0.01] rates (Fig. 1A-B). Multivariate analysis also showed that the DSS was associated with YY1 expression and that it could be used as an independent DSS indicator [HR = 1.84 (1.16, 2.91), **p < 0.01]. Hence, these results indicated the prognostic value of YY1 as a GC biomarker (Fig. 1C-D). Additionally, compared to adjacent normal tissues, the tumor tissues exhibited significantly higher YY1 expression. (t = 4.042, ***p < 0.001, Fig. 2A). Next, we performed bioinformatics analysis on YY1 using TCGA STAD dataset to clarify the underlying promotive mechanisms of YY1 on the invasion, metastasis, and drug resistance of GC cells. We detected 4386 significant DEGs between patients with high and low YY1 expression. Additionally, 105 transcriptional targets and 11 transcription factors related to YY1 were predicted using the TRRUST v2 database. Then, 4741 YY1 co-expression genes were retrieved from the cbioportal (Fig. 2B and D). Finally, 30 genes were identified in the intersection analysis between DEGs, co-expressed genes, regulators, and targets of YY1 (Venn diagram - Fig. 2E).

### Transferrin is a potential key protein regulated by YY1

To identify key genes potentially regulated by, or significantly correlated to YY1 and that presented the most important interactions with YY1, we performed a LASSO Cox analysis based on the 30 hub genes and YY1. The LASSO results led to YY1 and 5 YY1-related hub genes (Supplementary Figure A-B). The receiver operating characteristic (ROC) curve was used to predict the DSS of 1, 3, and 5 years and showed that this gene group might be used to predict the DSS in the cohort from TCGA (AUC of 1, 3, 5 years were 0.746, 0.747, and 0.701, respectively; Supplementary Figure C). Moreover, the expressions of VEGFB, DNAJB4 and Transferrin were positively correlated with YY1, while COX7C was negatively correlated with YY1 (Supplementary Figure D). The Kaplan-Meier analysis of TCGA STAD indicated that patients with high Transferrin expression were the most correlated to adverse DSS [^***^p < 0.01, HR = 2.17 (1.40–3.37)] and PFS [^**^p < 0.01, HR = 1.84 (1.28–2.65)] prognoses after surgery (Supplementary Figure E). A previous study has also suggested that Transferrin participates in systemic iron homeostasis. Our current results indicated that Transferrin is a potential key protein regulated by YY1.

### Overexpression of YY1 regulates the activity of the p53 signaling pathway and GC cell ferroptosis

We performed a KEGG enrichment analysis using the 30 hub genes. The KEGG analysis identified that the YY1-regulated ferroptosis was highly enriched in GC tumors, and transferrin was involved in the ferroptosis process (Fig. 2F-G). Furthermore, the GSEA showed that the TP53_ACTIVITY_THROUGH_PHOSPHORYLATION, SIGNALING_BY_TGFB_FAMILY_MEMBERS, and GLYCEROPHOSPHOLIPID_BIOSYNTHESIS were significantly enriched pathways regulated by YY1 (Fig. 2H). Altogether, these results indicated that the regulatory effect of YY1 on ferroptosis might be exerted through the p53 signaling pathway.

### Overexpression of YY1 might suppress the infiltration of immune cells in GC

**Fig. 3:**
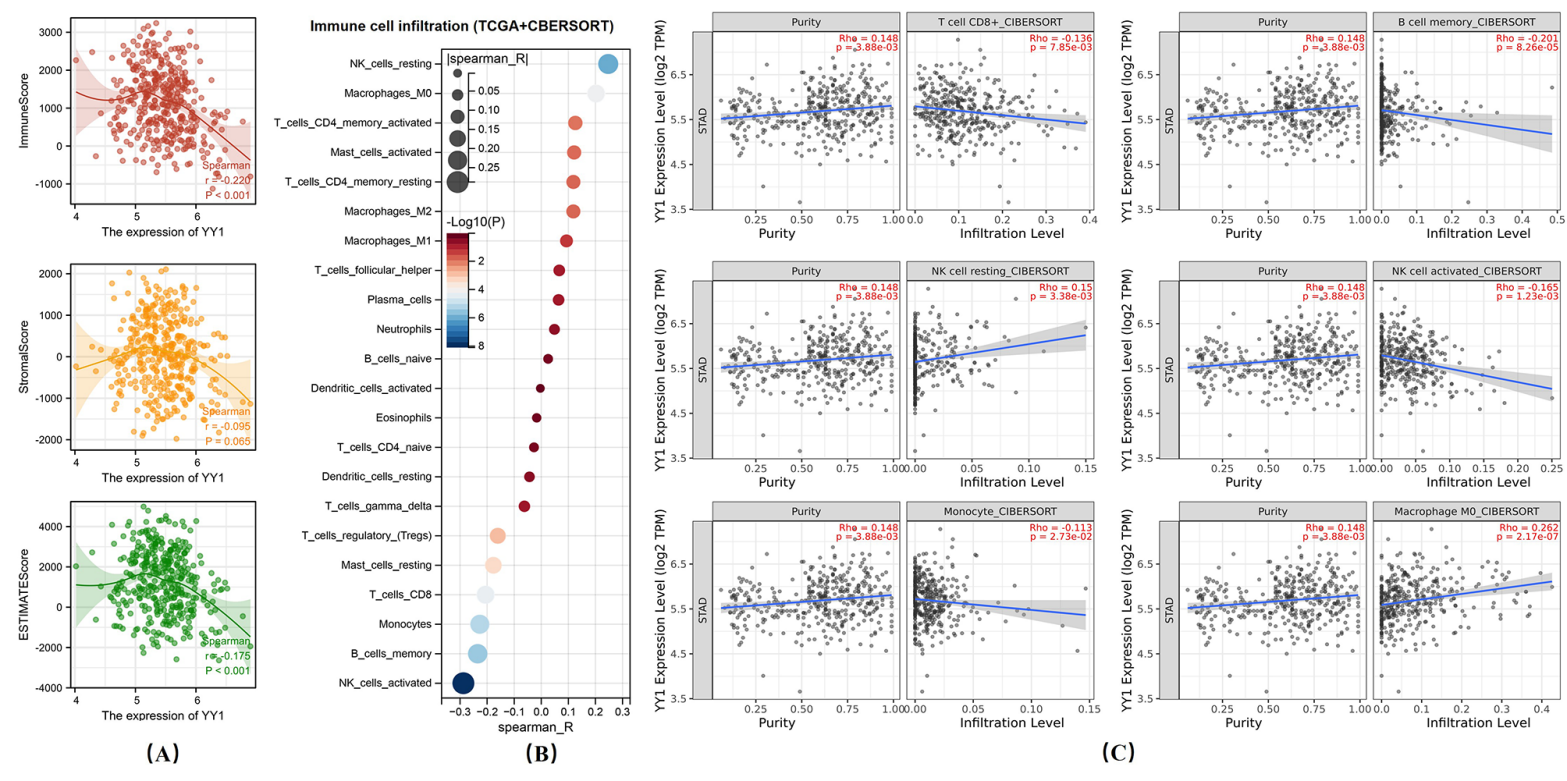
Overexpression of YY1 might suppress the infiltration of immune cells in GC. **(A)** YY1 expression was negatively correlated to IMMUNE (*r* = -0.220, *p* < 0.001), STROMAL (*r* = -0.095, *p* = 0.065), and ESTIMATE (*r* = -0.175, *p* < 0.001) scores. **(B)** CIBERSORT predicted that the expression of YY1 significantly suppressed immune cell infiltration. **(C)** The TIMER 2.0 showed that the infiltration of CD8 + T cells (Rho = -0.136, *p* < 0.001), B memory cells (Rho = -0.201, *p* < 0.001), active NK cells (Rho = -0.165, *p* < 0.001), and monocytes (Rho = -0.113, *p* < 0.001) was significantly reduced in tumor tissues, while the infiltration of NK resting cells (Rho = 0.15, *p* < 0.001) and macrophages (Rho = 0.262, *p* < 0.001) was enhanced

We explored the significance of the relationship between YY1 expression and the tumor immune microenvironment via the Spearman’s correlation coefficients between YY1 and immune infiltration. The expression of YY1 was negatively correlated with IMMUNE (*r* = -0.220, *p* < 0.001), STROMAL (*r* = -0.095, *p* = 0.065), and ESTIMATE (*r* = -0.175, *p* < 0.001) scores (Fig. 3A). The CIBERSORT analysis for TCGA STAD data showed that the expression of YY1 significantly suppressed immune cells infiltration. Finally, TIMER 2.0 verified that the infiltration of CD8 + T cells (Rho=-0.136, *p* < 0.001), B memory cells (Rho = -0.201, *p* < 0.001), active NK cells (Rho = -0.165, *p* < 0.001), and monocytes (Rho = -0.113, *p* < 0.001) was significantly reduced in tumor tissues, while the infiltration of NK resting cells (Rho = 0.15, *p* < 0.001) enhanced (Fig. 3B-C).

### Overexpression of YY1 inhibits GC cell ferroptosis and mediates Apatinib-resistance via the p53 signaling pathway

**Fig. 4:**
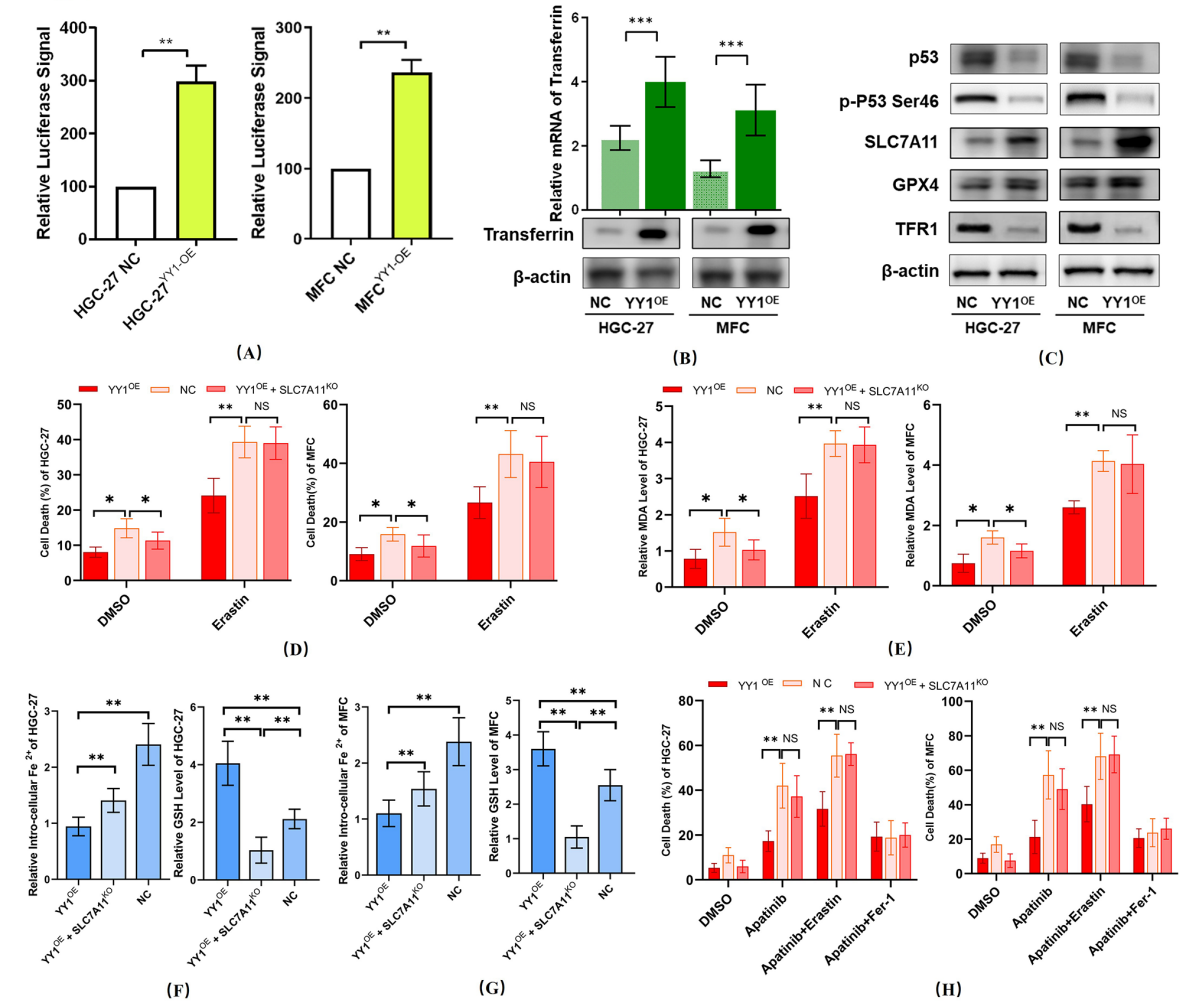
Overexpression of YY1 inhibits GC cell ferroptosis and mediates Apatinib-resistance via the p53 signaling pathway. **(A)** Luciferase reporter assay showed that Transferrin was transcriptionally regulated by YY1(*p < 0.05, t-test). **(B)**Western blot demonstrated that Transferrin expression was upregulated after YY1 overexpression. (C) Protein levels of p53 decreased after YY1 overexpression, furthermore SLC7A11 was upregulated, indicates YY1 inhibits gastric cancer via the p53 manner. Moreover, TFR1 was downregulated which might further inhibit GC cell ferroptosis. **(D-E)** YY1-overexpressed and YY1-overexpressed with SLC7A11 knock down HGC-27 and MFC cells were treated with Erastin in vitro. The cell death rate **(D)** and relative MDA levels **(E)** showed that YY1 overexpression could inhibit GC cell ferroptosis (*p < 0.05, ANOVA). **(F-G)** Relative intro-cellular Fe^2+^**(F)** and level intro-cellular GSH **(G)** level in YY1-overexpressed and YY1-overexpressed with SLC7A11 knock down HGC-27 and MFC cells (*p < 0.05, ANOVA). **(H)** YY1-overexpressed HGC-27 and MFC cells developed ferroptosis thus Apatinib drug resistance in vitro (*p < 0.05, ANOVA)

The bioinformatics results showed that Transferrin was a potential target regulated by YY1. Luciferase reporter assay showed that Transferrin was transcriptionally regulated by YY1 (Fig. 4A). Transferrin plays a complex role in the process of ferroptosis [27]. Some studies suggest that overexpression of transferrin triggers ferroptosis [28], while others indicate that Transferrin plays a role in systemic iron homeostasis [27, 29]. Our current GSEA showed that TP53_ACTIVITY_THROUGH_PHOSPHORYLATION was a significantly enriched pathway regulated by YY1. Previous literature reports that decreased p53 can promote the ubiquitination and degradation of TfR1 to inhibit ferroptosis, which may explain why in our study, TFR1 was downregulated via p53  signaling pathway, thus, the upregulated expression of Transferrin could not bind to TFR1 on the cell surface, thereby maintaining iron homeostasis and exerting an inhibitory effect on ferroptosis [29, 30]. Thus, our Western blot showed that YY1 overexpression directly upregulated Transferrin, inhibited p53 expression thus upregulated SLC2A11 (Fig. 4B-C), which might constitute the mechanism of ferroptosis inhibition after YY1 overexpression.

**Fig. 5:**
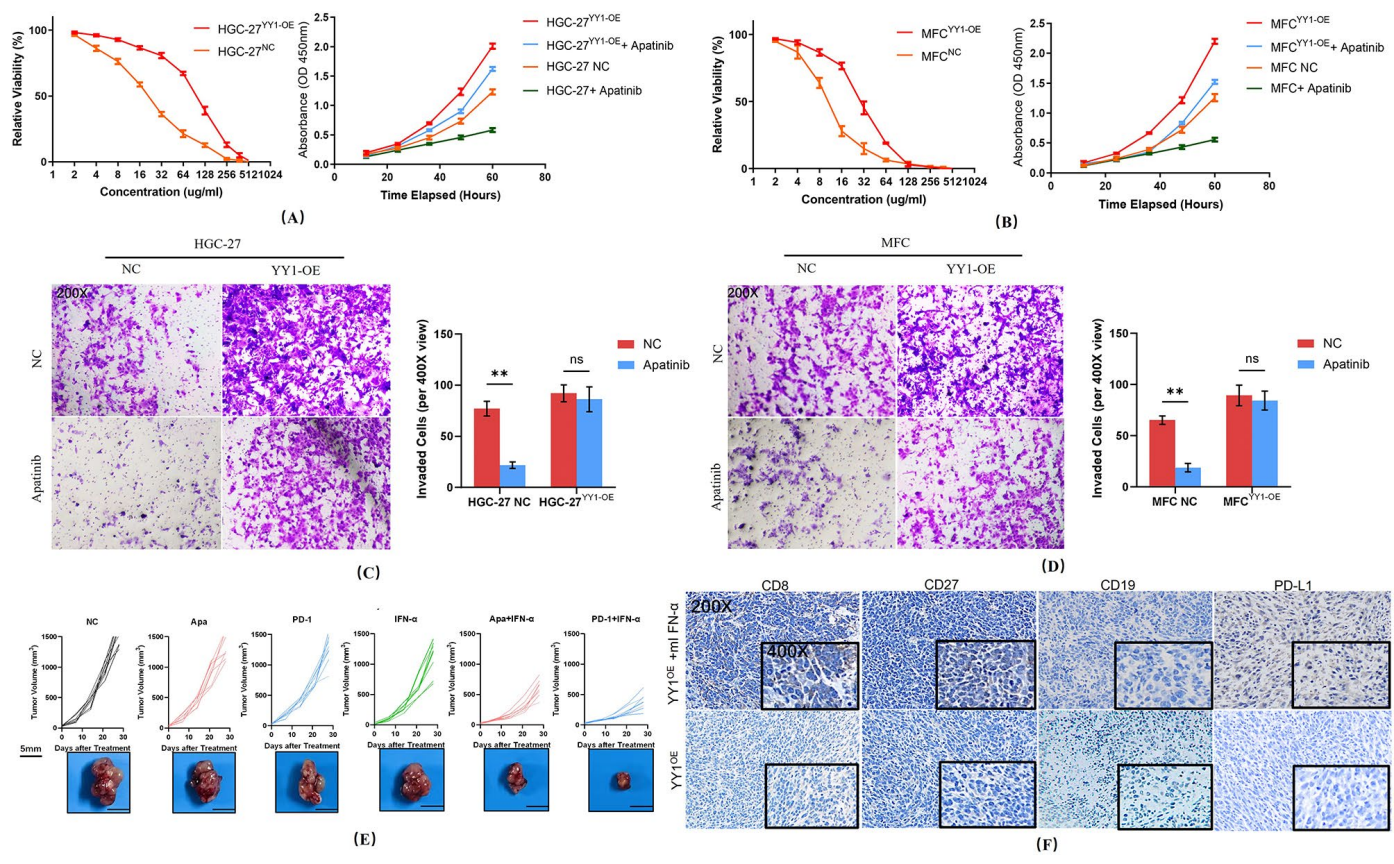
Effects of YY1 overexpression and knockdown on cell proliferation and by CCK-8 in HGC-27 and MFC cells. **(A-B)** After YY1 overexpression, the cell lines tumors presented a significant elevation on cell proliferation and drug resistance for Apatinib. The overexpression of YY1 led enhanced the invasion ability (**p < 0.001, non-paired t test) capacities by transwell assays in HGC-27 **(C)** and MFC **(D)** cells. **(E)** Effects of YY1 overexpression on tumor growth curve for subcutaneous tumors derived from cells infected with the lentivirus encoding YY1. After YY1overexpression, the subcutaneous tumors presented a significant drug resistance for both Apatinib and the mouse PD-1 antibody. The treatment with mouse IFN-α significantly reversed both Apatinib and mPD-1 antibody resistance of YY1 overexpressed subcutaneous tumors (**p < 0.001, Non-paired t test, Scale bar: 5 mm). **(F) **After treatment with mouse IFN-α, both CD8 and CD27 were significantly upregulated among GC tissues, indicating an improvement of the immune microenvironment of subcutaneous GC tissues (View: 200X and 400X)

To demonstrate the basis for YY1 in mediating ferroptosis and Apatinib resistance, YY1-overexpressed HGC-27 and MFC cells were treated with Fer-1, Erastin, Apatinib in vitro, cell death rate and relative MDA levels were also measured. First, YY1-overexpressed and YY1-overexpressed with SLC7A11 knock down HGC-27 and MFC cells were treated with Erastin in vitro. The cell death rate and relative MDA levels showed that YY1 overexpression could inhibit GC cell ferroptosis. Meanwhile, Erastin could reverse the inhibition effect (Fig. 4D). What’s more, loss of SLC7A11 could block the inhibition effect (Fig. 4E, *p < 0.05, ANOVA). Considering the effect of Transferrin in maintaining systemic iron homeostasis ferroptosis [27, 29, 31], we tested relative intro-cellular levels of Fe^2+^ and GSH. Relative GSH level in YY1-overexpressed HGC-27 and MFC cells was elevated (Fig. 4F *p < 0.05, ANOVA). However, Fe^2+^ maintains low level in YY1-overexpressed cell line and relative low level in YY1-overexpressed with SLC7A11 knock down cell line (Fig. 4G, *p < 0.05, ANOVA). YY1-overexpressed HGC-27 and MFC cells developed ferroptosis thus Apatinib drug resistance in vitro (Fig. 4H, *p < 0.05, ANOVA). Which shows inhibition of ferroptosis after YY1 overexpression via p53 signaling pathway and related to elevating Transferrin. Hence, the overexpression of YY1 could induce Apatinib drug resistance (Fig. 5A-B, IC50 of HGC-27: 80.94 vs. 20.74 ug/ml, IC50 of MFC: 27.09 vs. 10.52 ug/ml, Nonlin-Fit). Compared to normal control GC cells, YY1-overexpressed GC cells presented significantly enhanced growth, migration, and invasion. Thus, these results indicated the promotive effect of YY1 on GC cell growth, invasion, and metastasis (Fig. 5C-D).

Our previous study demonstrated that Interferon-α could remodel the hepatocellular microenvironment and potentiates anti-PD-1 efficacy [25]. These findings suggested that IFN-α might be an effective treatment for Apatinib and mPD-1-resistant GC cells.

To verify the promotive effect of YY1 on tumor growth in vivo, we injected YY1-overexpressed GC cells and measured the tumor progression. We found that overexpression of YY1 promoted tumor progression in vivo (Fig. 5E). Additionally, we analyzed the phenotypes of GC cells with YY1 overexpression after IFN-α treatment. The IFN-α treatment did not directly lead to decreased tumor growth in vivo, but significantly reverse both Apatinib and mPD-1 antibody resistance in YY1-overexpressed subcutaneous tumors (Fig. 5E). Finally, the IHC analysis demonstrated that, after treatment with mIFN-α, both CD8 and CD27 were significantly upregulated in GC tissues, thereby indicating an improvement in the immune microenvironment of GC tissues. However, CD19 and PD-L1 has no significant different between the two groups (Fig. 5F).

## Discussion

YY1 is a zinc finger protein that belongs to the Gli-Krüppel family [32]. It can act as an activator or repressor of gene transcription depending on the intracellular physiological state and microenvironment, as well as the presence of repression and activation domains at the C- and N-terminus, respectively. YY1 has been implicated in various biological processes, such as development, differentiation, cell cycle regulation, DNA repair, and apoptosis respectively, YY1 can act as an activator or repressor of gene transcription [33].

Previous studies have demonstrated the close association between YY1 expression and the prognosis of various cancers [34]. Additionally, YY1 can promote the proliferation and metastasis of GC via multiple cancer-related pathways [35–40]. YY1 can also induce immune therapy resistance through p53, miR34a, STAT3, NF-kB, PI3K/AKT/mTOR, c-Myc, and COX-2 [41]. Moreover, YY1 mRNA stabilization induced by HnRNP L can promote the transcription of PD-L1 in prostate cancer cell lines [42]. However, the role of YY1 in regulating PD-L1 expression and immune evasion in GC remains unclear [41].

In this study, we aimed to elucidate the molecular mechanisms and functional implications of YY1 in GC. We found that YY1 expression was significantly higher in GC tissues compared to adjacent normal tissues, and high YY1 expression was associated with poor disease-specific survival (DSS), indicating that YY1 may serve as an independent risk indicator for GC prognosis. Through bioinformatics analysis, we identified genes co-expressed with YY1 and pathways involved in YY1-mediated GC progression.

KEGG and GSEA analysis revealed a negative correlation between YY1 and ferroptosis-related genes in GC tissues. Ferroptosis is a form of regulated cell death dependent on intracellular iron concentrations and is associated with tumor growth and drug resistance. Our findings suggest that YY1 expression might inhibit GC ferroptosis, thereby mediating apatinib resistance and immune suppression.

Unlike autophagy, necrosis, and apoptosis, ferroptosis is dependent on intracellular iron concentrations and significantly associated with tumor growth and drug resistance. Ferroptosis is typically regulated by glutathione peroxidase 4 (GPX4) [43]. In 2015, Jiang et al. observed an association between inactivation of the p53 pathway and suppression of ferroptosis [44]. Regarding the relationship between Apatinib and ferroptosis, previous studies have shown that by causing lipid peroxidation via GPX4, Apatinib can negatively regulate GC cell ferroptosis [45]. Additionally, by suppressing VEGFR2/Nrf2/Keap1 activation and subsequent enhancement of ferroptosis, apatinib treatment significantly restrains the growth of glioma cells [46]. Apatinib might also enhance ELOVL6/ACSL4-mediated ferroptosis in colorectal cancer cells [47]. Hence, we hypothesized that YY1 expression might inhibit GC ferroptosis, thereby mediating apatinib resistance and immune suppression.

Our further experiments demonstrated that YY1 promoted GC cell growth and metastasis in vitro. Overexpression of YY1 decreased protein levels of p53, a tumor suppressor, and increased expression of SLC7A11, a key regulator of ferroptosis, indicating that YY1 inhibits GC cell ferroptosis via the p53 pathway. Moreover, TFR1 was downregulated through the p53 signaling pathway, thus the upregulated expression of transferrin could not bind to TFR1 on the cell surface, thereby maintaining iron homeostasis and exerting an inhibitory effect on ferroptosis. YY1 overexpression also conferred resistance to apatinib, a tyrosine kinase inhibitor known to trigger ferroptosis. These findings suggest that YY1 is a negative regulator of GC cell ferroptosis and a potential mediator of apatinib resistance. Additionally, YY1 overexpression led to increased intracellular levels of glutathione (GSH) and Fe^2+^, further supporting its role in inhibiting ferroptosis.

IFN-α is commonly used for the treatment of some cancer and viral diseases in clinical practice [48]. Meanwhile, IFN-α is widely used as a cancer therapeutic drug combined with novel strategies [48]. For instance, increased PD-L1 expression was observed after IFN-α administration in some human cancers, such as melanoma. However, combined with PD-1 blockade, IFN-α can boost powerful antitumor effects in B16 melanoma-bearing mice [49]. To overcome Apatinib resistance mediated by YY1, we tested the effect of mIFN-α, a type of interferon with anti-tumor and anti-viral activities, on YY1-overexpressed GC cells in vivo. We observed that mIFN-α treatment partially reversed YY1-mediated tumor growth and drug resistance by increasing the expression of CD8 and CD27, markers of T cell activation, in GC tissues. This suggests that YY1 may modulate the immune microenvironment of GC by affecting T cell-mediated immunity.

### Limitations

Our study offers valuable insights into the role of YY1 in the progression and prognosis of gastric cancer, yet it is not without its limitations. Predominantly, our conclusions are derived from bioinformatics analysis and experimental validation. While these methodologies are reliable, they may not fully capture the intricacies of the physiological environment within human patients. Additionally, while Transferrin was identified as a potential protein influenced by YY1, the exact dynamics and functional implications of this interaction in the context of GC progression require further investigation. Similarly, even though we determined that IFN-a can mitigate Apatinib resistance and immune suppression in GC tissues, the optimal dosage and potential adverse effects for human patients warrant further clinical trials. Future research should aim to address these limitations, thereby providing a more comprehensive understanding of YY1’s role and the therapeutic potential of IFN-a in gastric cancer.

## Conclusion

In conclusion, our study provides new insights into the role of YY1 in regulating ferroptosis and Apatinib resistance in GC. We demonstrate that YY1 acts as a negative regulator of GC cell ferroptosis through the p53 pathway and may contribute to Apatinib resistance. Furthermore, we propose a novel strategy to overcome this resistance and immune suppression by combining mIFN-α with PD-1 blockade. However, further studies are needed to fully understand the detailed molecular mechanisms of YY1 and IFN-α in GC. Additionally, clinical trials are required to evaluate the efficacy, safety, applicability, and dosage of this combination therapy in GC patients.

### Electronic supplementary material

Below is the link to the electronic supplementary material.

Supplementary Material 1. **Supplementary Figure** (A-B) The LASSO regression analysis of YY1 and YY1 expression-related hub genes, and LASSO coefficients of YY1 and 5 YY1-related hub genes, respectively. (C) The ROC curve for prognostic FRLS in TCGA STAD. (D) The expressions of VEGFB (Spearman *r* = 0.207, ***p < 0.001), DNAJB4(Spearman *r* = 0.131, *p = 0.011), CXCR4(Spearman *r* = 0.088, *p* = 0.090), and TF(Spearman *r* = 0.140, **p = 0.007) were positively correlated to YY1, while COX7C(Spearman *r*=-0.096, *p* = 0.063) was negatively correlated. (E) The Kaplan-Meier analysis of TCGA STAD indicated that patients with high TF(HR = 1.84[1.28–2.65], *p* = 0.001) expression have significantly adverse prognoses after surgery.

## Data Availability

The data that support the findings of this study are available in. https://www.gencodegenes.org/human/release_22/gencode.v22.annotation.gff3.gz. http://ftp.ebi.ac.uk/pub/databases/gencode/Gencode_human/release_33/gencode.v33.annotation.gff3.gz. http://ftp.ebi.ac.uk/pub/databases/gencode/Gencode_human. https://www.grnpedia.org/trrust/. https://www.kegg.jp/kegg/rest/keggapi.html. http://software.broadinstitute.org/gsea/index.jsp.
