# Supplementary figures and images for: YY1: a key regulator inhibits gastric cancer ferroptosis and mediating apatinib-resistance
Source: Cancer Cell Int. 2024 Feb 12;24:71. doi: 10.1186/s12935-024-03262-z (PMC10863212; doi:10.1186/s12935-024-03262-z)

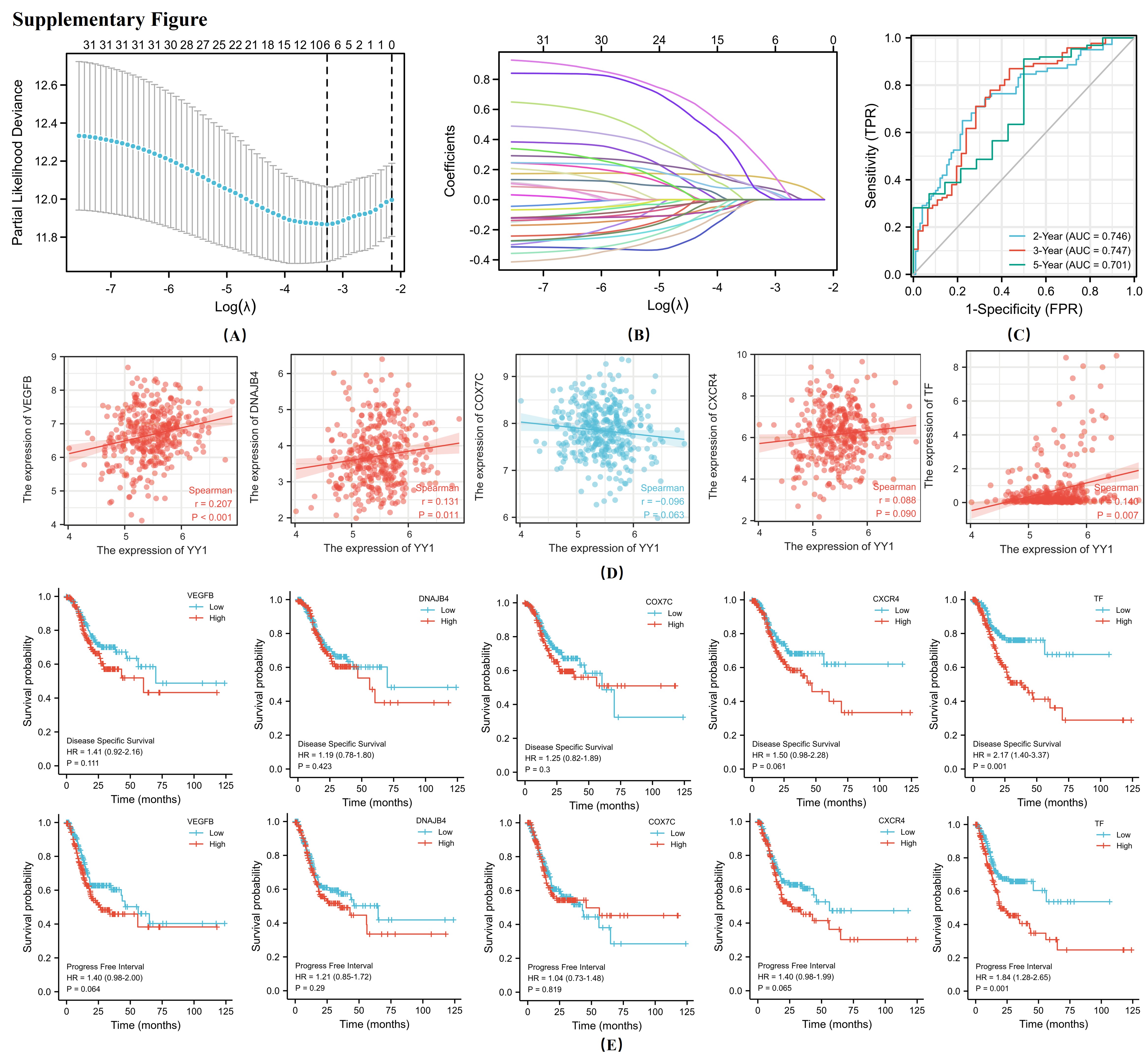

Supplement: Supplementary file 1 — Supplementary Material 1. Supplementary Figure (A-B) The LASSO regression analysis of YY1 and YY1 expression-related hub genes, and LASSO coefficients of YY1 and 5 YY1-related hub genes, respectively. (C) The ROC curve for prognostic FRLS in TCGA STAD. (D) The expressions of VEGFB (Spearman r = 0.207, ***p < 0.001), DNAJB4(Spearman r = 0.131, *p = 0.011), CXCR4(Spearman r = 0.088, p = 0.090), and TF(Spearman r = 0.140, **p = 0.007) were positively correlated to YY1, while COX7C(Spearman r=-0.096, p = 0.063) was negatively correlated. (E) The Kaplan-Meier analysis of TCGA STAD indicated that patients with high TF(HR = 1.84[1.28–2.65], p = 0.001) expression have significantly adverse prognoses after surgery. [file 12935_2024_3262_MOESM1_ESM.jpg]
